# Biomechanical investigation of two plating systems for medial column fusion in foot

**DOI:** 10.1371/journal.pone.0172563

**Published:** 2017-02-21

**Authors:** Paul Simons, Theresia Sommerer, Ivan Zderic, Dieter Wahl, Mark Lenz, Hristo Skulev, Matthias Knobe, Boyko Gueorguiev, R. Geoff Richards, Kajetan Klos

**Affiliations:** 1 Department of Foot and Ankle Surgery, Catholic Clinic Mainz, Mainz, Germany; 2 AO Research Institute Davos, Davos, Switzerland; 3 Department of Trauma, Hand and Reconstructive Surgery, University Hospital Jena, Jena, Germany; 4 Technical University Varna, Varna, Bulgaria; 5 Department of Trauma and Reconstructive Surgery, University Hospital Aachen, Aachen, Germany; Kanazawa University, JAPAN

## Abstract

**Background:**

Arthrodesis of the medial column (navicular, cuneiform I and metatarsal I) is performed for reasons such as Charcot arthropathy, arthritis, posttraumatic reconstruction or severe pes planus. However, the complication rate is still high and mainly resulting from inadequate fixation. Special plates, designed for medial column arthrodesis, seem to offer potential to reduce the complication rate. The aim of this study was to investigate biomechanically plantar and dorsomedial fusion of the medial column using two new plating systems.

**Methods:**

Eight matched pairs of human cadaveric lower legs were randomized in two groups and medial column fusion was performed using either plantar or dorsomedial variable-angle locking compression plates. The specimens were biomechanically tested under cyclic progressively increasing axial loading with physiological profile of each cycle. In addition to the machine data, mediolateral x-rays were taken every 250 cycles and motion tracking was performed to determine movements at the arthrodesis site. Statistical analysis of the parameters of interest was performed at a level of significance p = 0.05.

**Results:**

Displacement of the talo-navicular joint after 1000, 2000 and 4000 cycles was significantly lower for plantar plating (p≤0.039) while there was significantly less movement in the naviculo-cuneiform I joint for dorsal plating post these cycle numbers (p<0.001). Displacements in all three joints of the medial column, as well as angular and torsional deformations between the navicular and metatarsal I increased significantly for each plating technique between 1000, 2000 and 4000 cycles (p≤0.021). The two plating systems did not differ significantly with regard to stiffness and cycles to failure (p≥0.171).

**Conclusion:**

From biomechanical point of view, although dorsomedial plating showed less movement than plantar plating in the current setup under dynamic loading, there was no significant difference between the two plating systems with regard to stiffness and cycles to failure. Both tested techniques for dorsomedial and plantar plating appear to be applicable for arthrodesis of the medial column of the foot and other considerations, such as access morbidity, associated deformities or surgeon's preference, may also guide the choice of plating pattern. Further clinical studies are necessary before definitive recommendations can be given.

## Introduction

Arthrodesis of the navicular, cuneiform I and metatarsal I is performed for many reasons, such as Charcot arthropathy, arthritis, posttraumatic reconstructions or severe pes planus.

A variety of different foot arthrodesis techniques have been described in the past decades. External fixators, intramedullary midfoot fusion bolt systems, multiple screw fixations, medial or dorsomedial plating with or without compression screws have been regularly used [1–5]. Especially in neuropathic foot deformities a reasonable number of feet can be salvaged by arthrodesis when nonoperative treatments fail [1, 2, 5]. However, the current complication rate is still considerably high with up to 6 out of 7 patients needing revision surgery and it mainly results from inadequate stability of fixation [1, 3, 6].

Plantar plating of the tarsal bones has shown some clinical and biomechanical advantages [4, 7–9]. Therefore, special plates designed for medial column arthrodesis seem to offer potential to further reduce the complication rate in salvage surgery of the foot.

The aim of this study was to investigate whether there is a biomechanical benefit of plantar plating versus dorsomedial plating when arthrodesis of the medial column is performed. We hypothesized that plantar plating would provide substantial biomechanical benefits.

## Materials and methods

### Specimens and study groups

Eight pairs of fresh-frozen (-20 C°) human cadaveric lower legs from three female and five male donors aged 79.4 ± 11.8 years (mean ± standard deviation, range 59–91 years) were used in this study. All donors have given a signed agreement for scientific medical research and education during their lifetime. The specimens were provided by the Institute of Anatomy at the University Hospital Jena (Jena, Germany). Amputation was performed 6.5 cm below the mid tibia perpendicular to the tibial axis.

The specimens were allowed to defrost 24 hours at room temperature prior to preparation and biomechanical testing. Donors with diseases or medical history, that might have influenced bone structure, have been excluded. Radiographic evaluation prior to preparation ensured that there were no specimens with any bony deformities. The specimens were assigned pairwise to two study groups in a randomized manner for either plantar or dorsomedial plate arthrodesis.

### Surgical procedure

Both groups underwent arthrodesis by an experienced surgeon under fluoroscopic control according to the implant manufacturer's guidelines. A careful approach was performed in both groups. Joint articulations were left intact to minimize this type of disturbance in consistency with previous studies [4, 10, 11]. Screw length was selected individually. Care of the tibialis anterior and posterior tendons was taken during the whole procedure. If necessary, the plates were pre-contoured to the specimen's anatomy. Care was taken to bend the plates only between the screw holes. In both instrumentations the naviculo-cuneiform I joint and the first tarsometatarsal joint were compressed by using compression forceps and compression rods of the plating system according to the manufacturer's guidelines.

### Dorsomedial arthrodesis

Dorsomedial arthrodesis was performed using a 3.5 mm VA LCP Medial Column Fusion Plate with a length of 78 mm (DePuy Synthes, Zuchwil, Switzerland). A medial incision from the navicular down to the first metatarsal was performed. Three screws were inserted in the navicular, two screws in the cuneiform I and two screws in the metatarsal I.

### Plantar arthrodesis

Plantar arthrodesis was performed using a 3.5mm VA LCP Medial Column Fusion Plantar Plate with a length of 78 mm (DePuy Synthes, Zuchwil, Switzerland). The approach was performed by making an incision at the junction of the plantar and medial skin, right above the first metatarsal. Two screws were inserted in the navicular, the cuneiform I and the metatarsal I.

### Specimens preparation for biomechanical testing

The proximal 6.5 cm of the tibia and fibula of each specimen were embedded with polymethylmethacrylate (PMMA; Beracryl, Suter Kunststoff AG, Jegenstorf, Switzerland) in a 6.5 cm long cylindrical form with 4.8 cm diameter. The calcaneus was stripped of soft tissue. Care was taken to preserve the plantar aponeurosis, whereas the Achilles tendon was cut through. The periosteum was removed. A metallic tube clamp was wrapped around the calcaneus prior to embedding. An additional metallic tube clamp was wrapped around the PMMA to secure proper fitting. At a distance of 1 cm to the dorsal end of the PMMA embedding and corresponding to the tibial axis, a hole was drilled through the PMMA to fix a custom-made metallic turnbuckle as Achilles tendon equivalent.

### Biomechanical testing

**Fig 1:**
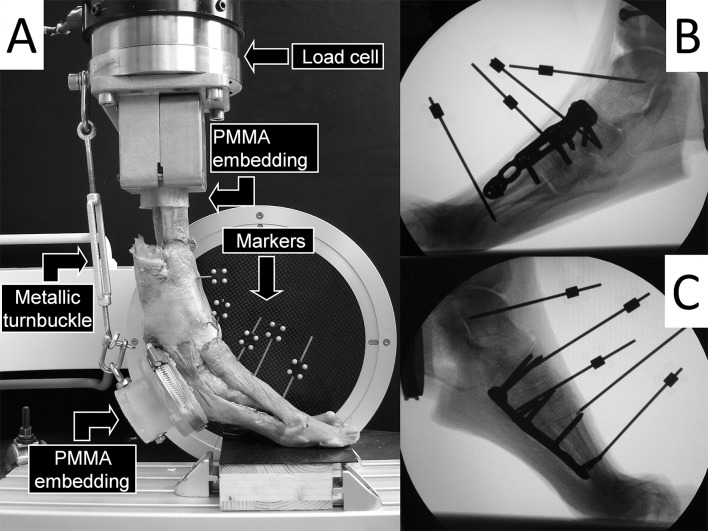
**A)** (left): Test setup with a left-foot specimen mounted for biomechanical testing and equipped with five retro-reflective marker sets for optical motion tracking, attached to the distal tibia, talus, navicular, cuneiform I and metatarsal I. The setup configuration allows full radiographic assessment by means of C-arm installed for mediolateral x-ray shots. **B)** (top right): Mediolateral exemplified x-ray of a specimen instrumented with dorsomedial fusion plate. **C)** (bottom right): Mediolateral exemplified x-ray of a specimen instrumented with plantar fusion plate.

The specimens were placed vertically on a servohydraulic testing machine (MTS Bionix 858; MTS Systems Corporation, Eden Prairie, MN) equipped with a 25 KN/200 Nm load cell. The setup with an examplified specimen mounted for biomechanical testing is shown in Fig 1A. The proximal specimen embedding was attached to the machine actuator. The toes were placed in a standardized, radiographically controlled manner on a custom-made radiolucent wooden platform. The calcaneus was attached to the machine actuator via the metallic turnbuckle, inserted into the hole of its PMMA embedding. The initial angle between the platform and the axis of metatarsal I was measured under 20 N axial compression preload. Then the metallic turnbuckle was tightened to achieve an inclination of 20 degrees more than this initial angle and an ankle knife to simulate forefoot weight bearing on the toes and the metatarsals. Five retro-reflective marker sets with four markers each were attached to the distal tibia, talus, navicular, cuneiform I and metatarsal I for optical motion tracking.

### Loading protocol

The loading protocol was adopted from previous studies performed by Rausch et al. [12] and Marks et al. [4]. It comprised initial quasi-static ramped compression loading from 20 N to 150 N at a rate of 13N/s, followed by cyclic axial compression at a rate of 2 Hz with progressively increasing loading and physiological profile of each cycle. Keeping the valley load at a constant level of 20 N, the peak load was constantly increased at a rate of 0.08 N/cycle, starting at 150N, until a catastrophic specimen failure occurred, or a test stop criterion was fulfilled. The latter was defined as 100 mm axial displacement of the machine actuator relative to its initial position under 20 N preload.

### Data acquisition and evaluation

Machine data in terms of axial displacement (mm) and axial load (N) were acquired from the machine actuator and the load cell at a rate of 128 Hz during biomechanical testing. Based on these, initial static stiffness (N/mm) was calculated from the linear slope of the axial load-displacement curve of the quasi-static ramp at the beginning of the test. Similarly, dynamic stiffness was evaluated at three different time points after 1, 1000 and 2000 cycles.

Moreover, mediolateral x-ray images of each specimen were taken for radiological assessment by the use of a triggered C-arm (Siemens ArcadisVaric, Siemens AG, Erlangen, Germany) at the beginning and the end of the quasi-static test, and then at timed intervals every 250 cycles during the cyclic test under peak load (Fig 1B and 1C). Based on this data, radiological failure was defined as bone fracture, screw pullout, implant loosening or a combination of these, and the corresponding cycles to radiological failure were calculated.

Optical motion tracking was performed to capture three-dimensional medial column joint movements at the arthrodesis site during biomechanical testing at a sampling rate of 100 Hz. Five ProReflex MCU digital cameras (Qualisys Motion Capture System; Qualisys AB, Gothenburg, Sweden) were used for this purpose. Based on this data, displacement at the dorsal aspect of the talo-navicular, naviculo-cuneiform I and cuneiform-metatarsal I joints, as well as angular and torsional deformation between navicular and metatarsal I after 1000, 2000 and 4000 cycles were calculated for all specimens.

In previous studies, construct failure was defined as displacement of 3 mm or more in any of the medial column joints [4, 13–15]. The numbers of cycles until fulfilment of this failure criterion for the dorsal aspect of each joint were calculated, resulting in the following three outcomes: cycles to 3 mm displacement at talo-navicular, cycles to 3 mm displacement at naviculo-cuneiform I and cycles to 3 mm displacement at cuneiform-metatarsal I joints. In addition, 10 degrees angular deformation between navicular and metatarsal I was defined as failure criterion and the respective cycles to fulfilment of this criterion calculated.

All parameters of interest selected for statistical analysis are summarised in Table 1.

**Table 1:** Overview of all parameters of interest selected for statistical analysis.

Source	Parameter of Interest
Machine data	Initial static stiffness
	Dynamic stiffness after 1 cycle
	Dynamic stiffness after 1000 cycles
	Dynamic stiffness after 2000 cycles
Motion tracking	Displacement at dorsal aspect of talo-navicular joint after 1000, 2000 and 4000 cycles
	Displacement at dorsal aspect of naviculo-cuneiform I joint after 1000, 2000 and 4000 cycles
	Displacement at dorsal aspect of cuneiform-metatarsal I joint after 1000, 2000 and 4000 cycles
	Angular deformation between navicular and metatarsal I after 1000, 2000 and 4000 cycles
	Torsional deformation between navicular and metatarsal I after 1000, 2000 and 4000 cycles
	Cycles to 3 mm displacement at talo-navicular, naviculo-cuneiform I and cuneiforn-metatarsal I joints
	Cycles to 10 degree angular deformation between navicular and metatarsal I

### Statistical analysis

Statistical analysis upon all parameters of interest was performed with the use of SPSS software package (IBM SPSS statistics V23, Armonk, NY, USA). Normal distribution of the data was screened with Shapiro-Wilk test. Statistical differences between the groups were assessed with Paired-Samples T-tests and General Linear Model Repeated Measures with Bonferroni Post Hoc Tests.

## Results

### Initial static stiffness

The initial static stiffness for the dorsomedial 3.5 mm VA-LCP Medial Column Fusion Plate was 17.9 ± 1.6 N/mm (mean ± standard error of mean, SEM). For the 3.5 mm VA-LCP Medial Column Fusion Plantar Plate the initial static stiffness was 18.3 ± 1.1 N/mm. Differences between the groups were not statistically significant (p = 0.736, S1 File).

### Dynamic stiffness after 1 cycle

The dynamic stiffness after 1 cycle for the dorsomedial plate construct was 34.3 ± 3.7 N/mm, whereas for the plantar plate construct it was 34.5 ± 3.2 N/mm, with no significant difference between the two implants (p = 0.927).

### Dynamic stiffness after 1000 cycles

The dynamic stiffness after 1000 cycles for the dorsomedial plate construct was 35.7 ± 3.6 N/mm, whereas for the plantar plate construct it was 38.1 ± 3.3 N/mm, with no significant difference between the two implants (p = 0.453).

### Dynamic stiffness after 2000 cycles

The dynamic stiffness after 2000 cycles for the dorsomedial plate construct was 39.5 ± 4.6 N/mm, whereas for the plantar plate construct it was 40.5 ± 3.9 N/mm, with no significant difference between the two implants (p = 0.636).

### Dynamic versus initial static stiffness

For each of the two plate constructs the initial static stiffness was significantly lower compared to the dynamic stiffness after 1, 1000 and 2000 cycles (p≤0.001). In addition, dynamic stiffness increased steadily but not significantly between cycles 1, 1000 and 2000 (p≥0.118).

### Optical motion tracking data

The results of the relative medial column joint movements investigated by means of three-dimensional optical motion tracking are summarised in Tables 2–4.

**Table 2:** Displacement at the dorsal aspect of the three measured joints in both groups with plantar and dorsomedial plating (mean ± standard error of mean, SEM, mm) after 1000, 2000 and 4000 cycles, together with p-values from the statistical analysis.

Joint	Groups	Displacement			P-value between groups
		1000 cycles	2000 cycles	4000 cycles	
Talo-navicular	Dorsal	1.79±0.40	2.91±0.66	5.23±1.16	0.039
	Plantar	1.53±0.37	2.60±0.63	3.38±0.92	
P-value between cycles		p < 0.001			
Naviculo-cuneiform I	Dorsal	0.31±0.12	0.49±0.18	1.78±0.60	<0.001
	Plantar	0.91±0.24	1.60±0.33	3.04±0.58	
P-value between cycles		0.001			
Cuneiform-metatarsal I	Dorsal	0.19±0.09	0.37±0.13	1.12±0.23	0.413
	Plantar	0.19±0.08	0.46±0.11	1.22±0.30	
P-value between cycles		<0.001			

**Table 3:** Angular and torsional deformation between navicular and metatarsal I in both groups with plantar and dorsomedial plating (mean ± standard error of mean, SEM, deg) after 1000, 2000 and 4000 cycles together with p-values from the statistical analysis.

Parameter	Groups	Deformation			P-value between groups
		1000 cycles	2000 cycles	4000 cycles	
Angular deformation	Dorsal	1.29±0.43	2.97±0.89	5.41±1.38	0.006
	Plantar	2.34±0.82	4.54±1.43	6.78±1.77	
P-value between cycles		<0.001			
Torsional deformation	Dorsal	0.99±0.37	1.84±0.68	3.34±1.17	0.637
	Plantar	1.10±0.34	2.15±0.76	3.97±1.91	
P-value between cycles		0.021			

**Table 4:** Failure criteria in both groups with plantar and dorsomedial plating, together with respective cycles to failure (mean ± standard error of mean, SEM) and p-values from the statistical analysis.

Failure criterion	Cycles to failure		p-value
	Dorsal	Plantar	
Radiological failure	9844±1572	8125±1434	0.255
3 mm displacement talo-navicular	3056±614	4111±1127	0.171
3 mm displacement naviculo-cuneiform I	8674±2481	5138±1350	0.206
3 mm displacement cuneiform-metatarsal I	10605±2308	9994±1888	0.791
10 deg angular deformation navicular-metatarsal I	8928±2493	6395±1497	0.327

**Fig 2:**
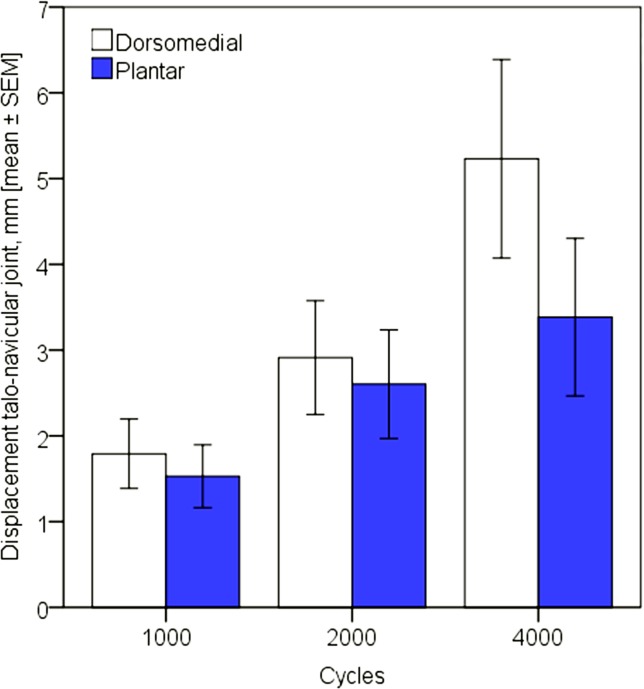
Displacement at the talo-navicular joint after 1000, 2000 and 4000 test cycles for dorsomedial and plantar plating.

**Fig 3:**
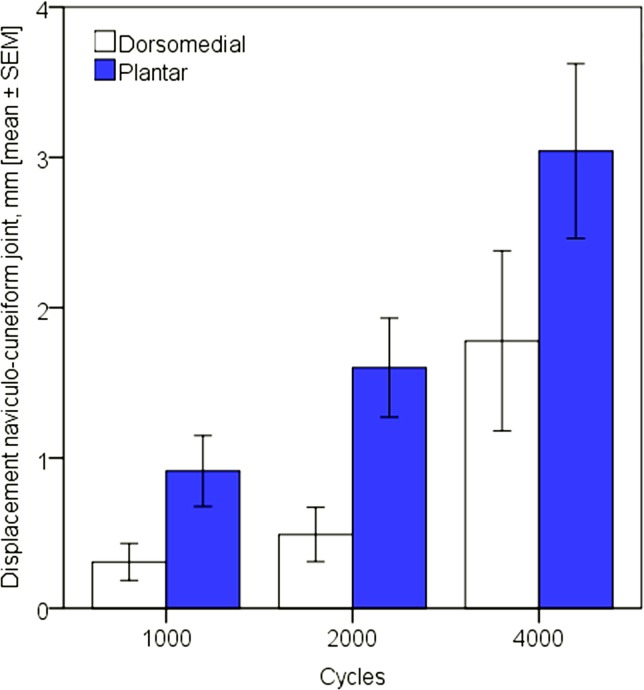
Displacement at the naviculo-cuneiform I joint after 1000, 2000 and 4000 test cycles for dorsomedial and plantar plating.

Displacements in all three joints, as well as angular and torsional deformations between navicular and metatarsal I increased significantly for each plating technique between 1000, 2000 and 4000 cycles, p≤0.021. Displacement at the talo-navicular joint after 1000, 2000 and 4000 cycles was significantly lower for plantar plating (p = 0.039) while there was significant less movement at the naviculo-cuneiform I joint for dorsal plating post these cycle numbers, p˂0.001 (Figs 2 and 3). Displacement at the cuneiform-metatarsal I joint was not significantly different between the two plate systems, p = 0.413. Similarly, torsional deformation between navicular and metatarsal I was comparable with no significant difference between the two plating techniques, p = 0.637, whereas angular deformation between these two bones was significantly bigger for plantar plating, p = 0.006.

There was no significant difference between the two implant systems with regard to cycles to failure with 3 mm displacement within any of the measured joints or 10 degree angular deformation between navicular and metatarsal I, p≥0.171.

### Cycles to radiological failure

Cycles to radiological failure were not significantly different between the two plating techniques, p = 0.255 (Table 4).

### Catastrophic failure type

With the exception of one specimen, radiological failure occurred in all tested specimens. No plate or screw breakage was observed. Screw loosening in the navicular was indicated in two of the tested specimens (12.5%). Cut-out of the navicular (defined as screws cutting through the bone) occurred in seven specimens (43.8%). Five out of the seven navicular cut-outs (71.4%) occurred in the specimens plated plantarly, whereas only two navicular fractures were seen in the group with dorsomedial plating (28.6%). Fracture of the metatarsal I appeared in two specimens (12.5%), whereas fracture of the metatarsal IV occurred in one specimen (6.3%). A fracture of the distal tibia was detected in one specimen (6.3%). Three specimens showed a calcaneus fracture (18.8%). Screw loosening in the metatarsal I appeared in two specimens (12.5%), screw loosening in the cuneiform I occurred in one specimen (6.3%). One specimen showed luxation of the talo-navicular joint as a radiological failure (6.3%).

## Discussion

The current study compared the biomechanical performance of two implant systems for arthrodesis of the navicular, cuneiform I and metatarsal I by means of either plantar or dorsomedial plating. These two methods of operative treatment become more and more prevalent with the increasing number of patients with neuropathic foot deformity [4].

To the best of our knowledge, neither biomechanical studies nor comparative clinical trials have been published so far, comparing dorsomedial versus plantar plate arthrodesis in this anatomical region.

Based on findings from previously published studies for tarsometatarsal I (TMT 1) arthrodesis, we hypothesized that plantar plate fixation would lead to a better biomechanical outcome [9]. However, this hypothesis could not be verified. What we did find instead was a decreased movement at the naviculo-cuneiform I joint and less angular deformation between the navicular and first metatarsal after dorsomedial plating, in contrast to less movement at the talo-navicular joint, which was not part of the arthrodesis site, after plantar plating.

From our perspective, the principle design of the dorsal implant might be one of the reasons for its better general performance in some aspects. This plate system seemed to enhance increased support for the navicular bone by its fixation with three variable-angle locking screws. In contrast, the plantar plating provided less contact area to this bone and fixed it with only two locking screws.

Moreover, we found that cut-out with fracturing of the navicular bone was the most common failure mode after plantar plating. Hence, it seemed like that the bone-screw interface at the navicular was the weakest part of this osteosynthesis.

The idea of plantar plating follows the principle of plate positioning on the tension side of the bony structures, aimed to provide better stability [14, 16]. However, a too rigid implant could counteract with this principle by increasing the peak stresses at the bone-screw interface and thus provoking screw cut-out and loosening. Theoretically, a less rigid and more forgiving plantar plate could have resulted in a more dynamic arthrodesis, enhancing better the tension-band effect with less cut-out rates. This statement is in line with the findings by Lill et al. that more rigid implants can lead to early loosening and failure at the bone-implant interface under dynamic loading, whereas implants with more elastic characteristics appear to minimize peak stresses at this interface, making them particularly suitable for fracture fixation in osteoporotic bone [17].

Following up the plantar plating, its observed decreased movement at the talo-navicular joint, which was not part of the medial column arthrodesis, could have resulted from the more expressed displacement at the medial naviculo-cuneiform joint registered after plantar plating, leading to less translated movement from this joint to the talo-navicular joint.

Despite the discussed contrasts, all other radiological and motion tracking indicators for primary and secondary stability, including cycles to failure and failure load did not exposed significant differences between the two techniques. Moreover, no implant failure in terms of plate or screw breakage was observed in the current study. These results support the findings that, despite some existing behavioral differences, both tested methods appear to be applicable for arthrodesis, which seems to incorporate enough biomechanical stability.

The outcomes from this study differ from previously reported results on arthrodesis of the TMT 1 joint by utilizing a plantar plate technique [9]. However, in contrast to this previous investigation we considered for arthrodesis not only the TMT 1 joint, but also included the medial naviculo-cuneiform joint. Moreover, whereas the previous study of isolated TMT 1 fusion utilized only bone models without any soft tissue, we used cadaveric lower legs with intact plantar soft tissue structures.

The current study has some limitations which are similar to those inherent to all cadaveric biomechanical studies. A limited number of specimens were used, thus restricting generalization to actual patients. In addition, the increasing bony union, that would have been expected to occur in vivo, could not be simulated. In agreement with Marks et al. and other previous authors [4, 18–22] we did not prepare the joint surfaces in order to eliminate one more variable which could have appeared from different preparation techniques. To support this argument, Ray et al. [10] have shown the impact of the different ways of articular surface preparation in a biomechanical study.

The current test setup was a gross simplification of in-vivo conditions. Our intention was to simulate strictly the conditions of the propulsion phase during walking. There can be no exact match with the in-vivo load applied until bony fusion is complete and our testing represented the worst-case scenario of an early full weight bearing mobilization without any protection. Postoperative mobilization is usually done with only partial weight bearing in a brace or a plaster cast and plantigrade loading.

Furthermore, although our test protocol simulated the force applied by the tension of the Achilles tendon, forces transferred by other tendons could not be taken into account, thus just partially reflecting real life conditions with the current biomechanical environment.

One of the main reasons for failed osteosynthesis or arthrodesis in patients could be the excessive repetitive loading over weeks and months. The application of progressively increasing cyclic loading aimed to achieve construct failure within a predefined number of cycles with specimens of different bone quality. The principle of testing with increasing load levels has proven to be useful in other biomechanical studies [23–25].

The donors' age was relatively advanced and therefore a reduced bone quality, similar to the bone quality of the affected population of patients, was expected even if the specimens in the current study were not evaluated for osteoporosis. For that reason, no quantitative statement about the bone quality could be made.

Whether the biomechanical behavior shown in the present study is clinically relevant and with impact in the clinics cannot be answered yet. Despite the absence of significant differences in most of the biomechanical parameters of interest, some other interesting factors need to be considered too. For instance, the plantar approach provides sufficient soft tissue coverage for a larger plate, however, its technical demands are higher than those for dorsomedial plating. Dorsomedial plating can help with reposition of the medial column and longer screws can be embedded in cuneiforms II and III to further stabilize the medial column.

## Conclusion

From biomechanical point of view, although dorsomedial plating showed less movement than plantar plating in the current setup under dynamic loading, there was no difference between the two plating systems with regard to stiffness and cycles to failure. Both tested techniques for dorsomedial and plantar plating appear to be applicable for arthrodesis of the medial column of the foot and other considerations, such as access morbidity, associated deformities or surgeon's preference, may also guide the choice of plating pattern. Further clinical studies are necessary before definitive recommendations can be given.

## Supporting information

S1 File Abstact presented at 'Foot International 2016' congress, June 23–25, 2016, Berlin, Germany. (PDF)
