# Supplementary material for: Biomechanical investigation of two plating systems for medial column fusion in foot
Source: PLoS One. 2017 Feb 21;12(2):e0172563. doi: 10.1371/journal.pone.0172563 (PMC5319781; doi:10.1371/journal.pone.0172563)
Supplement: S1 File — (PDF) [file pone.0172563.s001.pdf]

Parts of the study were presented at the "foot international" congress in Berlin 2016. The abstract of this presentation was published in a congress supplement of foot and ankle surgery under

http://www.footanklesurgery-journal.com/article/S1268-7731%2816%2930082-0/pdf

Abstract 6: Flatfoot / Foot and Ankle Surgery 22S (2016) 26–29

54

FI2016_Flatfoot_10

Biomechanical study on medial column fusion

K. Klos [1,*], T. Sommerer [2], Ivan Zderic [2], R.G. Richards [2], B. Gueorguiev [2], P. Simons [1]

[1] *Department of Foot and Ankle Surgery, Catholic Hospital Mainz, Germany*

[2] *AO Research Institute Davos, Switzerland*

*Corresponding author.

Background: Arthrodesis of the Os naviculare, Os cuneiforme I and Os metatarsale I (medial column) is performed for reasons such as Charcot or severe pes planus. There is a high complication rate mainly resulting from inadequate fixation. Special plates, designed for medial column arthrodesis, seem to offer potential to reduce the complication rate. The aim of this study was to compare biomechanically plantar and dorsomedial fusion using two new plating systems.

Materials: In eight matched pairs of human cadaveric legs medial column fusion was performed using either plantar or dorsomedial plate (VA;LCP Medial Column Fusion Plate; DePuy Synthes). Biomechanical testing was run under cyclic axial loading with physiological (Bergman) profile. AP X-rays were taken every 250 cycles. Motion tracking was applied to determine movements at the arthrodesis side.

Results: Angular and torsional deformations increased significantly in each group between 1000, 2000 and 4000 cycles. Displacement of the TN joint was significantly lower for plantar plating while there was significant less movement in the NC and TMT 1 joints for dorsal plating. The groups did not differ significantly with regard to stiffness and cycles to failure.

Conclusions: Dorsomedial plating showed less movement under cyclic loading in this setup. However, there was no difference between the two plating systems with regards to stiffness and cycles to failure. Thus, other considerations, such as access morbidity, associated deformities/ulcers or surgeon's preference, may also guide the choice of plating pattern. Further clinical studies are necessary before definitive recommendations can be given.

http://dx.doi.org/10.1016/j.fas.2016.05.060
